# Diagnostic utility of whole body Dixon MRI in multiple myeloma: A multi-reader study

**DOI:** 10.1371/journal.pone.0180562

**Published:** 2017-07-03

**Authors:** Timothy J. P. Bray, Saurabh Singh, Arash Latifoltojar, Kannan Rajesparan, Farzana Rahman, Priya Narayanan, Sahar Naaseri, Andre Lopes, Alan Bainbridge, Shonit Punwani, Margaret A. Hall-Craggs

**Affiliations:** 1 Centre for Medical Imaging, University College London, London, United Kingdom; 2 Cancer Research UK and UCL Clinical Trials Centre, London, United Kingdom; 3 Medical Physics Department, University College London Hospitals, London, United Kingdom; Henry Ford Health System, UNITED STATES

## Abstract

**Objective:**

To determine which of four Dixon image types [in-phase (IP), out-of-phase (OP), fat only (FO) and water-only (WO)] is most sensitive for detecting multiple myeloma (MM) focal lesions on whole body MRI (WB-MRI) images.

**Methods:**

Thirty patients with clinically-suspected MM underwent WB-MRI at 3 Tesla. Unenhanced IP, OP, FO and WO Dixon images were generated and read by four radiologists. On each image type, each radiologist identified and labelled all visible myeloma lesions in the bony pelvis. Each identified lesion was compared with a reference standard consisting of pre- and post-contrast Dixon and diffusion weighted imaging (read by a further consultant radiologist) to determine whether the lesion was truly positive. Lesion count, true positives, sensitivity, and positive predictive value were compared across the four Dixon image types.

**Results:**

Lesion count, true positives, sensitivity and confidence scores were all significantly higher on FO images than on IP images (p>0.05).

**Discussion:**

FO images are more sensitive than other Dixon image types for MM focal lesions, and should be preferentially read by radiologists to improve diagnostic accuracy and reporting efficiency.

## Introduction

In recent years, whole body-MRI (WB-MRI) has emerged as a valuable tool for assessing disease activity in multiple myeloma (MM).[1–5] MRI is a key component of the Durie-Salmon PLUS staging system[6], and the number of lesions identified on MRI correlates closely with mortality.[7] As a result, WB-MRI is developing into a first-line imaging modality in MM.[8,9]

The two major obstacles for widespread use of WB-MRI are cost and long scan times. It is therefore important to maximise diagnostic value but minimise acquisition time, particularly for MM patients who may be frail and in pain. To make best use of the available scan time, WB-MRI protocols typically include both anatomical imaging (for assessment of morphology, fractures and spinal cord compression[10,11]) and functional imaging (for assessing cellularity and perfusion[5,11–13]). However, imaging protocols vary substantially between centres: anatomical imaging may use T1-weighted or T2-weighted images (or a combination), and may implement spin echo- or gradient echo-based sequences.[14] When choosing sequences, considerations include image quality, acquisition time, the cost of data acquisition and storage, and interpretation time.

Recently, gradient echo-based Dixon MRI has been used for anatomical WB-MRI in MM[5,14,15], and has several advantages over conventional T1- or T2-weighted imaging. Dixon MRI enables the generation of four separate image types: in-phase (IP), out-of-phase (OP), water-only (WO) and fat-only (FO). Acquisition times are similar to those for conventional gradient echo imaging and shorter than for spin echo imaging.[15,16] When reporting, the IP images can be viewed in a similar fashion to conventional T1-weighted images, whilst water and fat can be separately evaluated on WO and FO images.

However, it is uncertain whether the ‘additional’ images (OP, WO and FO) offer any additional diagnostic information compared to IP imaging alone, and if so which image type is optimal for reading. Therefore, the best approach to reporting WB-MRI is unclear: it is uncertain whether reviewing the IP images alone is sufficient, or whether the additional images provide additional information. Clarifying this issue could improve the accuracy of disease staging and also increase reporting efficiency—radiologists could begin their read by reviewing the most diagnostically-useful scans. Furthermore, reconstructing and storing the additional images would only be justified if they provided additional diagnostic information.

In this study, we aimed to evaluate radiologists’ diagnostic accuracy for detecting focal lesions on each of the four Dixon image types, using post-contrast and diffusion images as a reference standard. We hypothesised that sensitivity would be improved by using FO and WO images compared to IP images.

## Materials and methods

### Subjects

This prospective study was performed with institutional review board approval (Research Ethics Committee reference 12/LO/0428). All patients gave written informed consent.

Thirty patients (13 males and 17 females, median age 55, age range 36–82) with clinically suspected symptomatic multiple myeloma were prospectively enrolled between June 2012 and September 2014. Patients were excluded if they had a history of previous. malignancy or previous chemotherapy/radiotherapy, estimated GFR < 50 mL/min/1.73 m^2^, were unable to given informed consent or had a contraindication to MRI scanning. Further assessment showed that 26 out of 30 had MM, one had smoldering MM, two a had solitary plasmocytoma, and one had monoclonal gammopathy of uncertain significance. For each patient, clinical and biochemical parameters were recorded as shown in Table 1. Baseline interphase fluorescence in situ hybridisation (FISH) was performed on CD138-selected plasma cells from bone marrow samples, using probes for IGH translocations t(4:14), t(11;14) and t(14;16), del(17p), del(13) and 1p-/1q+ [17]. Genetic risk was determined according to International Myeloma Working Group recommendations [18].

**Table 1:** Patient demographics, disease parameters and treatment. ISS, international staging system; DS-PLUS, PAD, bortezomib, doxorubicin, dexamethasone; CVD, cyclophosphamide, bortezomib, dexamethasone; VTD, bortezomib, thalidomide, bortezomib.dexamethasone; MPV, melphalan, prednisolone, bortezomib.

Patient characteristics	Number or median (range)
**Age (years)**	**56 (36–80)**
**Chain isotype**	
IgG	17
IgA	5
Light chain	4
MGUS	1
Solitary plasmacytoma	2
Smoldering MM	1
**ISS stage**	
I	13
II	13
III	4
**Induction regiment**	
PAD	18
CVD	3
VTD	5
MPV	2
**Bone marrow percentage plasma cells**	**65 (0–90)**
**Beta-2 microglobulin (mg/l)**	**3.3 (1.3–11.3)**
**Albumin (g/l)**	**40 (30–53)**
**Creatinine**	**56 (77.5–105)**
**Genetic risk group**	
Low/Standard risk	17
High risk	9

### Acquisition

All subjects underwent WB-MRI imaging on a 3.0T wide-bore system (Ingenia; Phillips Healthcare, Best, Netherlands) using two anterior surface coils, a head coil and an integrated posterior coil. The WB-MRI protocol included coronal pre- and post-contrast modified Dixon (Dixon) acquisitions from which fat and water images and calculated in and out of phase images were reconstructed on the scanner using a two-point method[19] (TR 3.0ms, TE 1.02–18, flip angle 15°, slice thickness 5mm, pixel bandwidth 1992Hz, acquisition matrix 196 x 238, SENSE factor 2, number of slice 120) in addition to diffusion and post-contrast imaging covering vertex to toe using ten contiguous anatomical stations (Table 2). The coronal images were ‘stitched’ together and presented as a head-to-toe whole body image to the reader; the images were then magnified according to the reader’s preference for specific analysis of the pelvis.

**Table 2:** Sequence parameters.

Sequence Parameters		
Parameters	Dixon (pre and post contrast)	DWI (b0, 100, 300, 1000 s/mm^2^)
**Imaging Plane**	Coronal	Transverse
**Sequence type**	Gradient echo	Single-shot spin echo with echo planar readout
**Echo time (ms)**	1.02/1.8	71
**Repetition time (ms)**	3	6371
**Field of View (mm x mm)**	502 x 300	500 x 306
**Voxel size (mm x mm)**	2.1 x 2.1	4 x 4.2
**Number of Slices**	120	40
**Slice Thickness (mm)**	5	5
**Acquisition Matrix**	144 x 238	124 x 72
**ETL**	2	39
**Acceleration factor (SENSE)**	2	2.5
**Pixel Bandwidth (Hz)**	1992	3369
**Scan time (s)**	17	152

### Image assessment

The individual sets of pre-contrast Dixon images were randomised and read by four consultant radiologists, who each had between five and fifteen years of specialist expertise in oncological MR imaging. All readers were blinded to clinical data and diagnosis. On each image set, each radiologist was asked to count the number of myeloma lesions present in the bony pelvis (pubis, ischium, ilium and sacrum) and to label these lesions on the images (up to a maximum of 20). If the disease was diffuse or there were over 20 lesions, the patient was assigned a lesion count of 20. Additionally, the radiologists were asked to provide a confidence score based on their degree of certainty that there were myeloma lesions in the pelvis on a 4-point Likert scale (1-no lesions, 2-indeterminate lesions, 3-likely myeloma lesions, 4-very likely myeloma lesions). After scoring, each labelled lesion was compared to a reference standard consisting of diffusion-weighted, pre- and post-contrast Dixon imaging, which had been evaluated by a further consultant radiologist with over 20 years of experience in myeloma and MR imaging. On the reference imaging, all lesions demonstrating abnormal marrow signal compared to background marrow (i.e. hypointense on IP and FO images, and hyperintense on WO images) and which showed contrast enhancement or restricted diffusion were assigned as myeloma lesions and labelled on the images. For the reference standard, no maximum lesion count was used (i.e. all lesions were labelled) to ensure that all lesions on the IP, OP, FO and WO Dixon images could be compared directly to a reference lesion. Using the reference standard imaging, we also recorded whether patients had focal or diffuse disease (the diffuse category included patients with focal-on-diffuse infiltration).

For each Dixon image set, we compared each lesion with the reference standard to determine the number of per-set true positive lesions (TP), false positive lesions (FP) (i.e. those that were incorrectly identified as lesions); and false negative lesions (FN) (these were the ‘reference-standard lesions’ which were not identified). For each Dixon image type (30 sets per type), we determined the mean per-set lesion count, sensitivity (TP/TP+FN), positive predictive value (TP/TP + FP) and mean confidence score.

### Design and statistics

**Fig 1:**
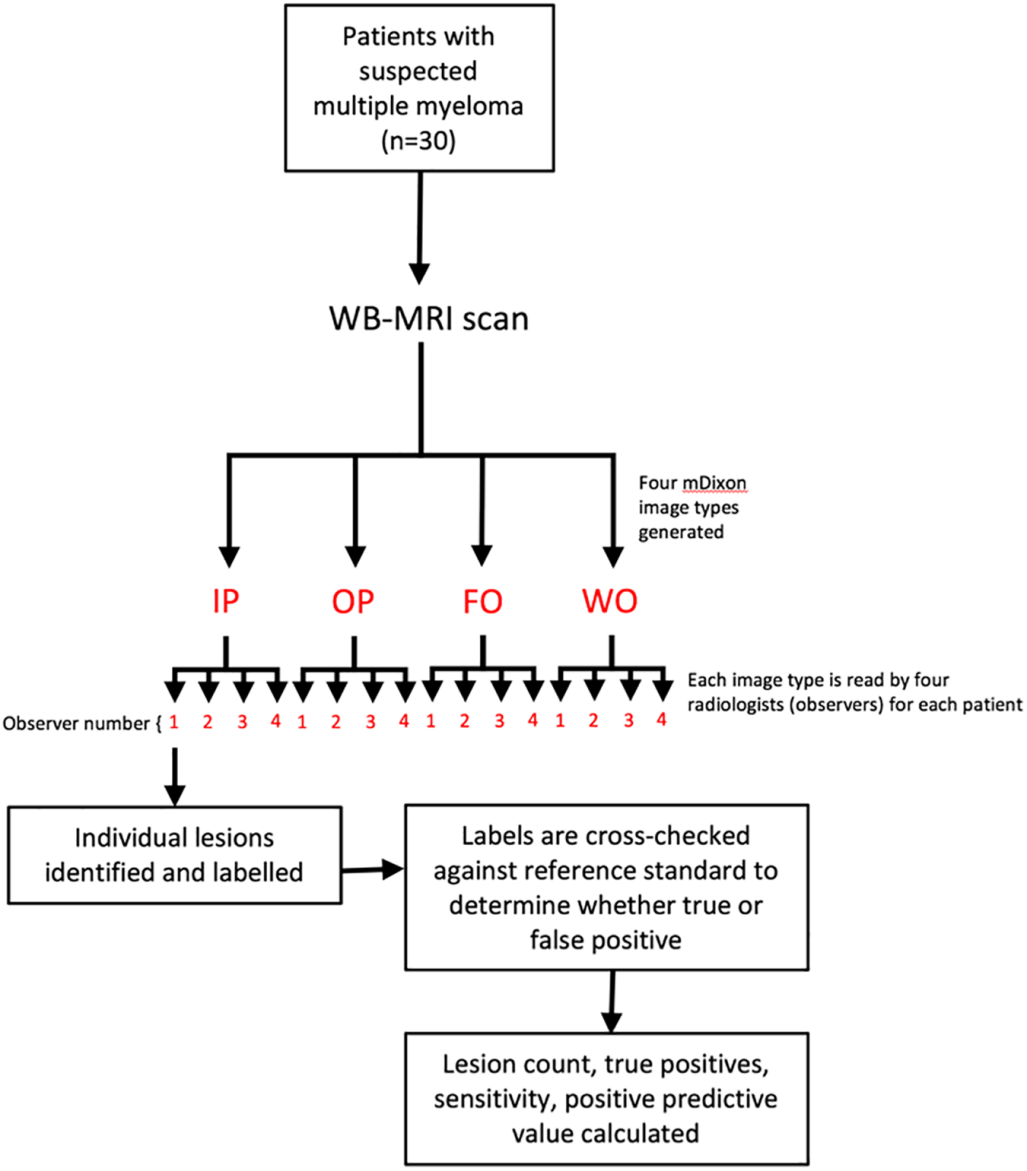
Study design.

A summary of the study design is given in Fig 1. To account for clustering within the data, for each lesion detection metric (lesion count, sensitivity, positive predictive value and mean confidence score), values were compared across the four Dixon image types using a multilevel mixed-effects linear regression model, performed using Stata [Stata IC Version 14.1, College Station, USA]. Image type (i.e. IP, OP, FO or WO) was used as the predictor variable, and the value of the specific lesion detection metric being analysed (i.e. lesion count, sensitivity, positive predictive value or mean confidence score) was used as the outcome variable. Data were clustered at the level of ‘subject’ (patient) and ‘observer’ (radiologist). This analysis was repeated for the subgroup of patients who had diffuse disease (as determined by the reference standard assessment), and for the subgroup of patients with focal disease.

### Percent contrast and contrast-to-noise ratio

Percent contrast and contrast-to-noise ratio (CNR) were calculated using a previously described method [15]. Specifically, in patients with at least three focal lesions greater than 3mm in diameter, circular regions of interest (ROIs) were placed on the three largest focal myeloma lesions, and three further ROIs were placed in areas of bone marrow without focal lesions in the sacrum and iliac bones.

Percent contrast was calculated as:

$$Percent\;Contrast=\frac{\left(S_{a}-S_{b}\right)}{\left(S_{a}+S_{b}\right)}$$

where *S*_*a*_ is the mean signal intensity of myeloma lesions and *S*_*b*_ is the background marrow signal intensity.

Similarly, CNR was calculated as:

$$CNR=\frac{|S_{a}-S_{b}|}{\sqrt{(S_{asd}+S_{bsd})/2}}$$

where *S*_*asd*_ and *S*_*bsd*_ are the mean within-ROI standard deviation values for myeloma lesions and background marrow respectively. A one-way analysis of variance (ANOVA) with a post-hoc Tukey Kramer multiple comparison test was used to compare percent contrast and CNR between image series.

## Results

**Fig 2:**
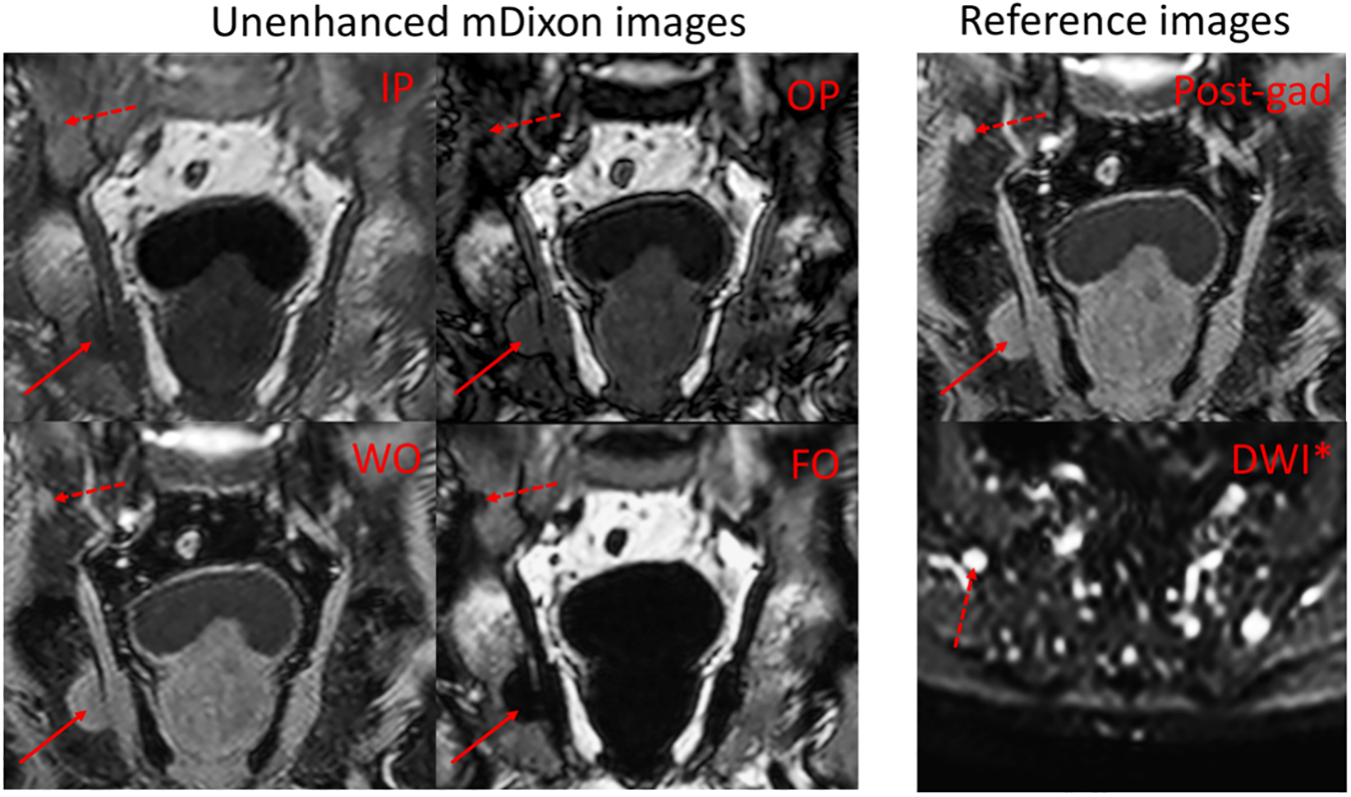
Examples of focal MM lesions. There is a large focal lesion in the right ischium (solid arrow) and a smaller lesion in the right ilium (dashed arrow); both lesions are shown on unenhanced Dixon images (IP, OP, WO and FO) and on the reference images (consisting of DWI and post-contrast). The smaller lesion is less conspicuous on the IP and OP images than on the FO and WO images. *DWI was acquired in the axial plane; the smaller of the two lesions (in the right ilium) is again marked with a dashed arrow.

**Fig 3:**
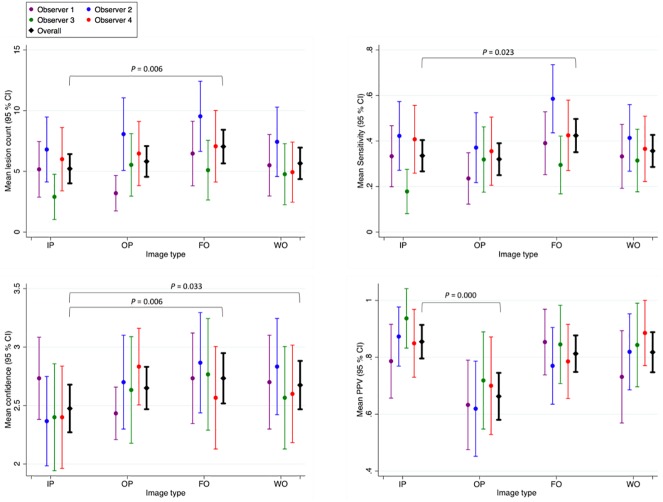
Lesion count, sensitivity, positive predictive value (PPV) and confidence for each Dixon image type. Individual observers are shown in colour (see legend), and the mean value across all four observers is shown in black. Error bars indicate the 95% confidence interval.

Four radiologists read four image series for each of 30 patients (120 image series per radiologist), and identified 610, 955, 549 and 734 lesions respectively compared to 1560 reference lesions. An example of a focal lesion, as shown on the four Dixon image types, is given in Fig 2. A summary of the mean lesion count, true positives, sensitivity, positive predictive value and confidence score for each of the four image types is given in Table 3; these values are also shown graphically in Fig 3. The results of the regression analysis including confidence intervals are also provided in Table 3.

**Table 3:** Results of regression analysis (n = 30).

**Lesion Count**			
**Image type**	**Mean**	**Difference in means (95% CI)**	**p-value**
**IP**	**5.2**	**Baseline**	**-**
**OP**	**5.8**	**+0.60 (-0.70 to +1.90)**	**0.364**
**FO**	**7.0**	**+1.83 (+0.53 to +3.12)**	**0.006**
**WO**	**5.7**	**+0.44 (-0.85 to +1.74)**	**0.504**
**True positives**			
**Image type**	**Mean**	**Difference in means (95% CI)**	**p-value**
**IP**	**4.9**	**Baseline**	**-**
**OP**	**4.6**	**-0.29 (-1.48 to +0.90)**	**0.633**
**FO**	**6.5**	**+1.63 (-1.48 to +0.90)**	**0.008**
**WO**	**5.1**	**+0.23 (-0.96 to +1.43)**	**0.702**
**Sensitivity**			
**Image type**	**Mean**	**Difference in means (95% CI)**	**p-value**
**IP**	**0.34**	**Baseline**	**-**
**OP**	**0.32**	**-0.02 (-0.09 to +0.06)**	**0.696**
**FO**	**0.42**	**+0.09 (+0.01 to +0.16)**	**0.023**
**WO**	**0.36**	**+0.02 (-0.06 to +0.10)**	**0.590**
**Positive predictive value**			
**Image type**	**Mean**	**Difference in means (95% CI)**	**p-value**
**IP**	**0.86**	**Baseline**	**-**
**OP**	**0.67**	**-0.17 (-0.24 to -0.10)**	**0.000**
**FO**	**0.81**	**-0.05 (-0.12 to +0.02)**	**0.146**
**WO**	**0.82**	**-0.02 (-0.09 to + 0.05)**	**0.617**
**Confidence Score (/4)**			
**Image type**	**Mean**	**Difference in means (95% CI)**	**p-value**
**IP**	**2.48**	**Baseline**	**-**
**OP**	**2.65**	**+0.18 (-0.01 to +0.36)**	**0.063**
**FO**	**2.73**	**+0.26 (+0.07 to +0.44)**	**0.006**
**WO**	**2.68**	**+0.20 (+0.16 to + 0.38)**	**0.033**

Lesion count, true positives, sensitivity, positive predictive value and confidence were compared between the four image types, using the in phase images as the baseline. Regression analyses used image type were used as the predictor variable, and lesion count/TP/sensitivity/confidence were used as the outcome variable. Mean values were calculated by the regression analysis, and were equal to means calculated manually from all patients and all four radiologists.

### Lesion count and true positives

The mean lesion counts for each image type (averaged over all patients and all four radiologists) were 5.2 for IP, 5.8 for OP, 7.0 for FO and 5.7 for WO. Significantly more lesions were identified on the FO images than on the IP images (p = 0.006), but there was no significant difference between OP and IP images (p = 0.364) or WO and IP images (p = 0.504).

Of the identified lesions, the mean number of true positives was 4.9 for IP, 4.6 for OP, 6.5 for FO and 5.1 for WO. Significantly more true positive lesions were identified on the FO images than on the IP images (p = 0.008), but there was no significant difference in true positives between OP and IP images (p = 0.633) or WO and IP images (p = 0.702).

### Sensitivity and positive predictive value

The mean sensitivity for each image type was 0.34 for IP, 0.32 for OP, 0.42 for FO and 0.36 for WO. Sensitivity was significantly higher on the FO images than on the IP images (p = 0.023), but there was no significant difference between OP and IP images (p = 0.696) or between WO and IP images (p = 0.590).

The mean positive predictive values were 0.86 for IP, 0.67 for OP, 0.81 for FO and 0.82 for WO. There was no significant difference in PPV for FO compared to IP (p = 0.146) or WO compared to IP (p = 0.617). However, positive predictive values were significantly poorer on OP images than on IP images (p = 0.000).

### Confidence score

The mean confidence scores were 2.48 for IP, 2.65 for OP, 2.73 for FO and 2.68 for WO (1-no lesions, 2-indeterminate lesions, 3-likely myeloma lesions, 4-very likely myeloma lesions). Confidence scores were higher on all three image types than on the IP images (OP compared to IP: p = 0.063, FO compared to IP: p = 0.006, WO compared to IP: p = 0.033).

### Sub-group analysis

Of 30 patients, there were 23 patients in the focal disease group (this included the two patients with solitary plasmacytoma) and six patients in the diffuse disease group. True positives, sensitivity and PPV for focal and diffuse groups are given in Table 4 and Table 5 respectively.

**Table 4:** Results of regression analysis for the focal lesion group alone (n = 23).

**True positives**			
**Image type**	**Mean**	**Difference in means (95% CI)**	**p-value**
**IP**	**5.1**	**Baseline**	**-**
**OP**	**5.0**	**-0.08 (-1.38 to +1.21)**	**0.900**
**FO**	**6.8**	**+1.77 (+0.47 to +3.07)**	**0.008**
**WO**	**5.2**	**+0.11 (-1.19 to +1.42)**	**0.863**
**Sensitivity**			
**Image type**	**Mean**	**Difference in means (95% CI)**	**p-value**
**IP**	**0.37**	**Baseline**	**-**
**OP**	**0.36**	**-0.00 (-0.09 to +0.08)**	**0.976**
**FO**	**0.46**	**+0.09 (-0.01 to +0.18)**	**0.037**
**WO**	**0.38**	**+0.02 (-0.07 to +0.10)**	**0.689**
**Positive predictive value**			
**Image type**	**Mean**	**Difference in means (95% CI)**	**p-value**
**IP**	**0.79**	**Baseline**	**-**
**OP**	**0.63**	**-0.14 (-0.21 to -0.07)**	**0.000**
**FO**	**0.78**	**-0.02 (-0.09 to +0.05)**	**0.516**
**WO**	**0.72**	**-0.00 (-0.08 to +0.07)**	**0.936**

True positives, sensitivity, positive predictive value and confidence are compared across the four image types, using the in phase images as the baseline.

**Table 5:** Results of regression analysis for the diffuse disease group (n = 6).

**True positives**			
**Image type**	**Mean**	**Difference in means (95% CI)**	**p-value**
**IP**	**4.0**	**Baseline**	**-**
**OP**	**2.9**	**-1.13 (-4.0 to +1.8)**	**0.449**
**FO**	**5.0**	**+1.04 (-1.9 to +4.0)**	**0.483**
**WO**	**4.7**	**+0.71 (-2.2 to +3.6)**	**0.634**
**Sensitivity**			
**Image type**	**Mean**	**Difference in means (95% CI)**	**p-value**
**IP**	**0.22**	**Baseline**	**-**
**OP**	**0.15**	**-0.07 (-0.23 to +0.09)**	**0.398**
**FO**	**0.29**	**+0.08 (-0.09 to +0.24)**	**0.349**
**WO**	**0.25**	**+0.03 (-0.12 to +0.19)**	**0.674**
**Positive predictive value**			
**Image type**	**Mean**	**Difference in means (95% CI)**	**p-value**
**IP**	**0.85**	**Baseline**	**-**
**OP**	**0.58**	**-0.27 (-0.46 to -0.09)**	**0.004**
**FO**	**0.65**	**-0.15 (-0.33 to +0.035)**	**0.113**
**WO**	**0.85**	**-0.06 (-0.26 to +0.13)**	**0.522**

True positives, sensitivity, positive predictive value and confidence are compared across the four image types, using the in phase images as the baseline.

In the focal disease group, true positives and sensitivity were significantly higher in the FO group than in the IP group (p = 0.008 and 0.037 respectively). There was no significant difference in PPV between IP and FO groups (p = 0.516). The OP images performed significantly less well than IP images in terms of PPV (p = 0.000).

In the diffuse disease group, there were no significant differences between FO and IP groups in terms of true positives, sensitivity or PPV (p = 0.483, p = 0.349 and p = 0.113 respectively). PPV was again significantly poorer on the OP images than on the IP images (p = 0.004).

True positives and sensitivity were higher for the focal disease group (sensitivity on FO images was 0.29 in the diffuse group and 0.46 in the focal group), although these groups were not formally compared.

### Percent contrast and contrast-to-noise ratio

**Fig 4:**
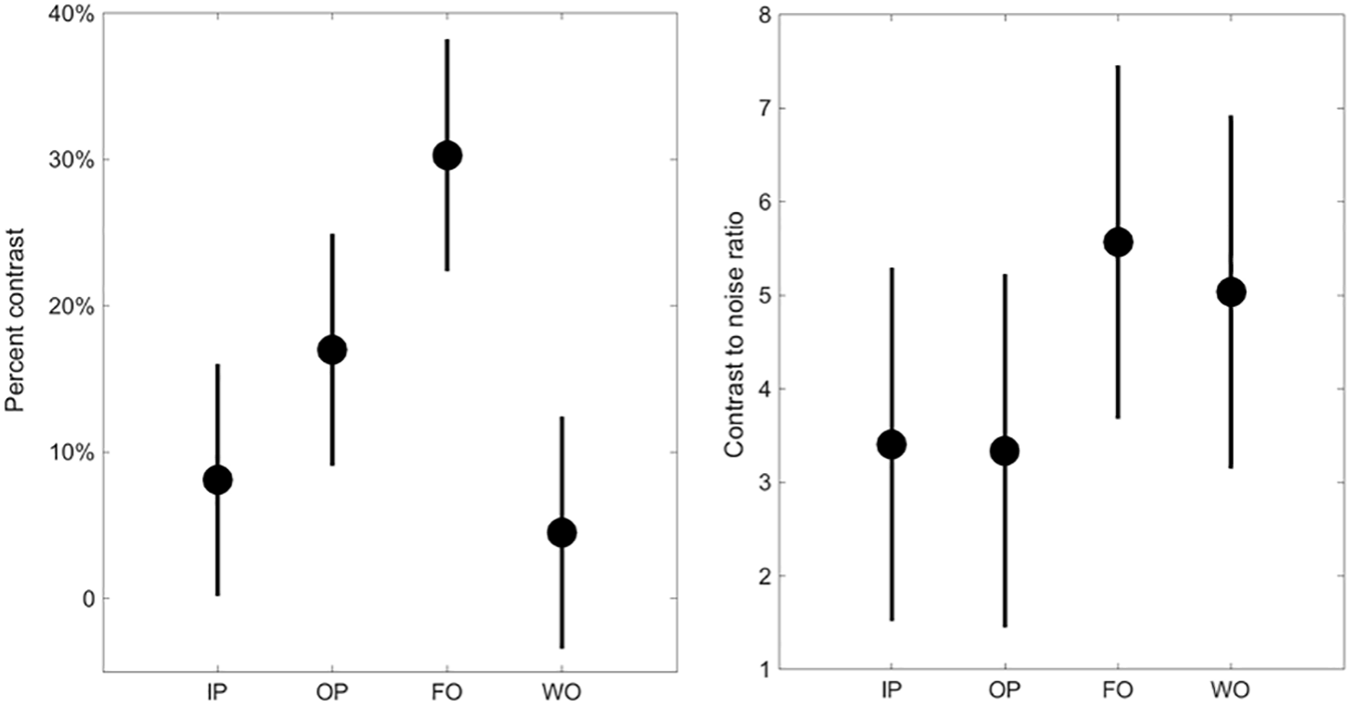
Comparison of percent contrast and CNR between groups. The figures show the results of a post-hoc multiple comparison test from a one-way ANOVA. Estimates of Percent Contrast and CNR are shown as circles; the comparison intervals for each group are shown as solid lines. Percent contrast was significantly higher on FO images than on IP images (p = 0.003).

Comparison of percent contrast and CNR between groups is demonstrated in Fig 4. Percent contrast was highest in the FO group (the values for each image type were, IP: 8.1, OP: 17, FO: 30 and WO: 4.5), and was significantly higher for FO images than for IP images (p = 0.003). There was no significant difference between OP and IP images, or between WO and IP images.

Contrast to noise ratio was also highest in the FO group (the values for each image type were, IP: 3.41, OP: 3.34, FO: 5.57 and WO: 5.04). However, was no significant difference in CNR between groups.

## Discussion

In this study, lesion counts, true positive counts, sensitivity, positive predictive value and reader confidence were compared across the four Dixon images types. We have shown that FO images are superior to other image types and in particular IP images in terms of lesion counts, true positives, sensitivity and confidence. Furthermore, our data suggest that focal lesions demonstrate greater contrast compared to background marrow on FO images than on IP images, which may account for the superior sensitivity of FO images. The positive predictive values for FO images were similar to those for IP and WO images and higher than those for OP images, suggesting that the increase in sensitivity reflects a true increase in lesion conspicuity rather than a lower reader threshold for lesion identification. The use of FO images offered the greatest advantage for patients with focal lesions, but also provided superior sensitivity in patients with diffuse disease.

The superior performance of FO imaging could occur because myelomatous infiltration of the bone marrow causes a proportionally greater reduction in marrow fat content than in water content. Normal adult bone marrow typically consists of 50–90% fat[20–22] and infiltration with myeloma cells decreases fat content[13,23,24]; however, the increase in water content may be relatively less because myeloma cells have an increased nuclear to cytoplasmic ratio.[25] This suggestion is supported by the observation that focal lesions are more difficult to detect in younger patients with cellular bone marrow imaging [26] or in myeloma patients with a higher bone marrow cell percentage.[15]

To our knowledge, this is the first study comparing lesion detection rates on individual Dixon images in patients with MM. A small number of studies have examined lesion contrast in Dixon imaging compared to other sequences[15,27], but none of these have directly examined lesion detection rates by radiologists. This study suggests that the use Dixon imaging improves diagnostic sensitivity and confidence compared to in phase T1-weighted gradient echo imaging alone. We therefore argue that Dixon imaging should be used in preference to T1-weighted imaging alone for anatomical WB-MRI in MM. Furthermore, radiologists should specifically review the FO image type when reading WB-MRI in MM to increase diagnostic yield and improve reporting efficiency.

The accuracy of lesion detection in MM directly impacts on assessment of disease burden and therefore prognosis.[7] Walker et al. showed that patients with more than seven focal lesions on WB-MRI had a five year survival of 55%, compared to 73% for those with no focal lesions [7]. Moulopolos et al. similarly showed that radiological assessment of disease burden could be used to separate patients into different survival categories.[28] In patients with only a small number of lesions, poor diagnostic sensitivity could theoretically alter the diagnosis itself–small volume disease could be missed altogether, or patients with a small number of lesions (>1) could be incorrectly diagnosed with solitary plasmacytoma.

A limitation of this study is that our observations are confined to images generated using a single Dixon sequence. It would be preferable to compare sensitivity and positive predictive value across gradient echo (Dixon) and spin echo images including T1-weighted and STIR images, to form a more definitive overall assessment of the optimal sequence. However, this type of study would be difficult to perform in practice since acquiring conventional T1-weighted spin echo images in addition to Dixon images would be extremely time consuming. Furthermore, previous studies suggest that gradient echo imaging offers similar image quality spin echo imaging in MM.[16]

The study is also limited by the nature of the scoring system used. In particular, the upper limit of 20 for the lesion count means that we have not captured differences in the number of lesions detected in patients with very high tumour load. However, the clinical importance of these differences is doubtful and current staging systems do not differentiate between patients with more than 20 lesions.[6,29] Our scoring system also penalises observers who fail to identify diffuse infiltration, leading to generally low sensitivity scores when compared to the reference standard.

Further work is required to examine the diagnostic utility of different MR sequences to arrive at an optimised protocol for WB-MRI in MM. In particular, it would be useful to determine the extent to which DWI, post-contrast and pre-contrast Dixon imaging each contribute to the overall interpretation of the WB-MRI scan. Careful assessment of the ‘value’ of each sequence is essential if cost-effective, high volume whole body scanning is to be achieved. High-value MRI is becoming an increasingly important goal for the imaging community[30], and studies specifically examining the value of WB-MRI in MM will be essential for widespread clinical implementation.

## Conclusion

Fat-only Dixon images offer higher lesion detection rates compared to in-phase images alone in multiple myeloma. We suggest that radiologists should preferentially review the fat-only images when reading to improve diagnostic accuracy and reporting efficiency.

## Supporting information

S1 File Raw data showing lesion detection rates for the four Dixon image types. Lesion counts, true positives, false positives, false negatives, sensitivity, PPV and confidence are provided for each of the image types, for each observer and each patient. Please refer to the Materials and Methods section for more information on data arrangement.
(XLSX)

## References

[1] International Myeloma Working Group. Criteria for the classification of monoclonal gammopathies, multiple myeloma and related disorders: a report of the International Myeloma Working Group. Br J Haematol. 2003;121(5):749–57. 12780789

[2] Dimopoulos M, Terpos E, Comenzo RL, Tosi P, Beksac M, Sezer O, et al International myeloma working group consensus statement and guidelines regarding the current role of imaging techniques in the diagnosis and monitoring of multiple Myeloma. Leuk Off J Leuk Soc Am Leuk Res Fund, UK. 2009;23(9):1545–56. 10.1038/leu.2009.89 19421229

[3] Dimopoulos M, Kyle R, Fermand JP, Rajkumar SV, San Miguel J, Chanan-Khan A, et al. Consensus recommendations for standard investigative workup: Report of the International Myeloma Workshop Consensus Panel 3. In: Blood. 2011. p. 4701–5. 10.1182/blood-2010-10-299529 21292778

[4] Bird JM, Owen RG, D’Sa S, Snowden JA, Pratt G, Ashcroft J, et al Guidelines for the diagnosis and management of multiple myeloma 2011. Br J Haematol. 2011;154(1):32–75. doi: 10.1111/j.1365-2141.2011.08573.x 21569004 10.1111/j.1365-2141.2011.08573.x

[5] Giles SL, Messiou C, Collins DJ, Morgan VA, Simpkin CJ, West S, et al Whole-Body Diffusion-weighted MR Imaging for Assessment of Treatment Response in Myeloma. Radiology [Internet]. 2014;271(3):131529 Available from: http://www.ncbi.nlm.nih.gov/pubmed/24475858 10.1148/radiol.13131529 24475858

[6] Durie BGM, Kyle R a, Belch A, Bensinger W, Blade J, Boccadoro M, et al Myeloma management guidelines: a consensus report from the Scientific Advisors of the International Myeloma Foundation. Hematol J. 2003;4:379–98. doi: 10.1038/sj.thj.6200312 14671610

[7] Walker R, Barlogie B, Haessler J, Tricot G, Anaissie E, Shaughnessy JD, et al Magnetic resonance imaging in multiple myeloma: diagnostic and clinical implications. J Clin Oncol. 2007 3;25(9):1121–8. doi: 10.1200/JCO.2006.08.5803 17296972 10.1200/JCO.2006.08.5803

[8] Rajkumar SV, Dimopoulos MA, Palumbo A, Blade J, Merlini G, Mateos M-V, et al International Myeloma Working Group updated criteria for the diagnosis of multiple myeloma. Lancet Oncol. 2014 11;15(12):e538–48. doi: 10.1016/S1470-2045(14)70442-5 25439696 10.1016/S1470-2045(14)70442-5

[9] Excellence NI of H and C. Myeloma: diagnosis and management. NG35. 2016;(February).

[10] Lecouvet FE, Vande Berg BC, Michaux L, Malghem J, Maldague BE, Jamart J, et al Stage III multiple myeloma: clinical and prognostic value of spinal bone marrow MR imaging. Radiology. 1998;209(3):653–60. doi: 10.1148/radiology.209.3.9844655 9844655 10.1148/radiology.209.3.9844655

[11] Lecouvet FE. Whole-Body MR Imaging: Musculoskeletal Applications. Radiology. 2016;279(2):345–65. doi: 10.1148/radiol.2016142084 27089188 10.1148/radiol.2016142084

[12] Messiou C, Collins DJ, Morgan VA, Desouza NM. Optimising diffusion weighted MRI for imaging metastatic and myeloma bone disease and assessing reproducibility. Eur Radiol. 2011;21(8):1713–8. doi: 10.1007/s00330-011-2116-4 21472473 10.1007/s00330-011-2116-4

[13] Zamagni E, Cavo M. The role of imaging techniques in the management of multiple myeloma. Br J Haematol. 2012 12;159(5):499–513. doi: 10.1111/bjh.12007 22881361 10.1111/bjh.12007

[14] Dixon WT. Simple proton spectroscopic imaging. Radiology. 1984;153(1):189–94. doi: 10.1148/radiology.153.1.6089263 6089263 10.1148/radiology.153.1.6089263

[15] Takasu M, Tamura T, Kaichi Y, Tanitame K, Akiyama Y, Date S, et al Magnetic resonance evaluation of multiple myeloma at 3.0 Tesla: how do bone marrow plasma cell percentage and selection of protocols affect lesion conspicuity? PLoS One. 2014 1;9(1):e85931 doi: 10.1371/journal.pone.0085931 24489680 10.1371/journal.pone.0085931 PMC3904853

[16] Weckbach S, Michaely HJ, Stemmer A, Schoenberg SO, Dinter DJ. Comparison of a new whole-body continuous-table-movement protocol versus a standard whole-body MR protocol for the assessment of multiple myeloma. Eur Radiol. 2010 12;20(12):2907–16. doi: 10.1007/s00330-010-1865-9 20574630 10.1007/s00330-010-1865-9

[17] Smith D, Stephenson C, Percy L, Lach A, Chatters S, Kempski H, et al Cohort analysis of FISH testing of CD138+ cells in relapsed multiple myeloma: Implications for prognosis and choice of therapy. Br J Haematol. 2015;171(5):881–3. doi: 10.1111/bjh.13446 25899469 10.1111/bjh.13446

[18] Sonneveld P, Avet-Loiseau H, Lonial S, Usmani S, Siegel D, Anderson KC, et al Treatment of multiple myeloma with high-risk cytogenetics: A consensus of the International Myeloma Working Group. Blood. 2016;127(24):2955–62. doi: 10.1182/blood-2016-01-631200 27002115 10.1182/blood-2016-01-631200 PMC4920674

[19] Eggers H, Brendel B, Duijndam A, Herigault G. Dual-Echo Dixon Imaging with Flexible Choice of Echo Times. 10.1002/mrm.22578 20860006

[20] Karampinos DC, Melkus G, Baum T, Bauer JS, Rummeny EJ, Krug R. Bone marrow fat quantification in the presence of trabecular bone: Initial comparison between water-fat imaging and single-voxel MRS. Magn Reson Med. 2014;71(3):1158–65. doi: 10.1002/mrm.24775 23657998 10.1002/mrm.24775 PMC3759615

[21] Li X, Kuo D, Schafer AL, Porzig A, Link TM, Black D, et al Quantification of vertebral bone marrow fat content using 3 Tesla MR spectroscopy: Reproducibility, vertebral variation, and applications in osteoporosis. J Magn Reson Imaging. 2011;33(4):974–9. doi: 10.1002/jmri.22489 21448966 10.1002/jmri.22489 PMC3072841

[22] Liney GP, Bernard CP, Manton DJ, Turnbull LW, Langton CM. Age, gender, and skeletal variation in bone marrow composition: A preliminary study at 3.0 Tesla. J Magn Reson Imaging. 2007;26(3):787–93. doi: 10.1002/jmri.21072 17729356 10.1002/jmri.21072

[23] Takasu M, Tani C, Sakoda Y, Ishikawa M, Tanitame K, Date S, et al Iterative decomposition of water and fat with echo asymmetry and least-squares estimation (IDEAL) imaging of multiple myeloma: initial clinical efficiency results. Eur Radiol [Internet]. 2012;22(5):1114–21. Available from: http://www.ncbi.nlm.nih.gov/pubmed/22138735 doi: 10.1007/s00330-011-2351-8 22138735 10.1007/s00330-011-2351-8

[24] Takasu M, Kaichi Y, Tani C, Date S, Akiyama Y, Kuroda Y, et al Iterative decomposition of water and fat with echo asymmetry and least-squares estimation (IDEAL) magnetic resonance imaging as a biomarker for symptomatic multiple myeloma. PLoS One. 2015;10(2):e0116842 doi: 10.1371/journal.pone.0116842 25706753 10.1371/journal.pone.0116842 PMC4338220

[25] D’Onofrio G, Zini G. Morphology of Blood Disorders. Morphology of Blood Disorders. 2014. 1–787 p.

[26] Hillengass J, Weber M-A, Kilk K, Listl K, Wagner-Gund B, Hillengass M, et al Prognostic significance of whole-body MRI in patients with monoclonal gammopathy of undetermined significance. Leukemia. 2014 1;28(1):174–8. doi: 10.1038/leu.2013.244 23958921 10.1038/leu.2013.244

[27] Costelloe CM, Madewell JE, Kundra V, Harrell RK, Bassett RL, Ma J. Conspicuity of bone metastases on fast Dixon-based multisequence whole-body MRI: clinical utility per sequence. Magn Reson Imaging. 2013 6;31(5):669–75. doi: 10.1016/j.mri.2012.10.017 23290478 10.1016/j.mri.2012.10.017 PMC3648589

[28] Moulopoulos LA, Gika D, Anagnostopoulos A, Delasalle K, Weber D, Alexanian R, et al Prognostic significance of magnetic resonance imaging of bone marrow in previously untreated patients with multiple myeloma. Ann Oncol. 2005 11;16(11):1824–8. doi: 10.1093/annonc/mdi362 16087694 10.1093/annonc/mdi362

[29] Kyle RA, Child JA, Anderson K, Barlogie B, Bataille R, Bensinger W, et al Criteria for the classification of monoclonal gammopathies, multiple myeloma and related disorders: A report of the International Myeloma Working Group. Vol. 121, British Journal of Haematology. 2003 p. 749–57. 12780789

[30] ISMRM. The MR Value Initiative [Internet]. Available from: http://www.ismrm.org/the-mr-value-initiative-phase-1/

